# *Angiostrongylus cantonensis* induces energy imbalance and dyskinesia in mice by reducing the expression of melanin-concentrating hormone

**DOI:** 10.1186/s13071-024-06267-9

**Published:** 2024-04-23

**Authors:** Hui Huang, Zhongyuan Zhang, Mengdan Xing, Zihan Jin, Yue Hu, Minyu Zhou, Hang Wei, Yiwen Liang, Zhiyue Lv

**Affiliations:** 1 Key Laboratory of Tropical Disease Control, Ministry of Education, Sun Yat-Sen University, Guangzhou, Guangdong Province 510030 People’s Republic of China; 2 Department of Pathogen Biology and Biosafety, Zhongshan School of Medicine, Sun Yat-Sen University, Guangzhou, Guangdong Province 510030 People’s Republic of China; 3 Hainan General Hospital, Hainan Affiliated Hospital of Hainan Medical University, Haikou, Hainan Province 570311 People’s Republic of China

**Keywords:** *Angiostrongylus cantonensis*, Melanin-concentrating hormone, Energy balance, Dyskinesia, Lateral hypothalamus, Neuroprotection

## Abstract

**Background:**

Infection with *Angiostrongylus cantonensis* (AC) in humans or mice can lead to severe eosinophilic meningitis or encephalitis, resulting in various neurological impairments. Developing effective neuroprotective drugs to improve the quality of life in affected individuals is critical.

**Methods:**

We conducted a Gene Ontology enrichment analysis on microarray gene expression (GSE159486) in the brains of AC-infected mice. The expression levels of melanin-concentrating hormone (MCH) were confirmed through real-time quantitative PCR (RT–qPCR) and immunofluorescence. Metabolic parameters were assessed using indirect calorimetry, and mice’s energy metabolism was evaluated via pathological hematoxylin and eosin (H&E) staining, serum biochemical assays, and immunohistochemistry. Behavioral tests assessed cognitive and motor functions. Western blotting was used to measure the expression of synapse-related proteins. Mice were supplemented with MCH via nasal administration.

**Results:**

Postinfection, a marked decrease in *Pmch* expression and the encoded MCH was observed. Infected mice exhibited significant weight loss, extensive consumption of sugar and white fat tissue, reduced movement distance, and decreased speed, compared with the control group. Notably, nasal administration of MCH countered the energy imbalance and dyskinesia caused by AC infection, enhancing survival rates. MCH treatment also increased the expression level of postsynaptic density protein 95 (PSD95) and microtubule-associated protein-2 (MAP2), as well as upregulated transcription level of B cell leukemia/lymphoma 2 (*Bcl2*) in the cortex.

**Conclusions:**

Our findings suggest that MCH improves dyskinesia by reducing loss of synaptic proteins, indicating its potential as a therapeutic agent for AC infection.

**Graphical Abstract:**

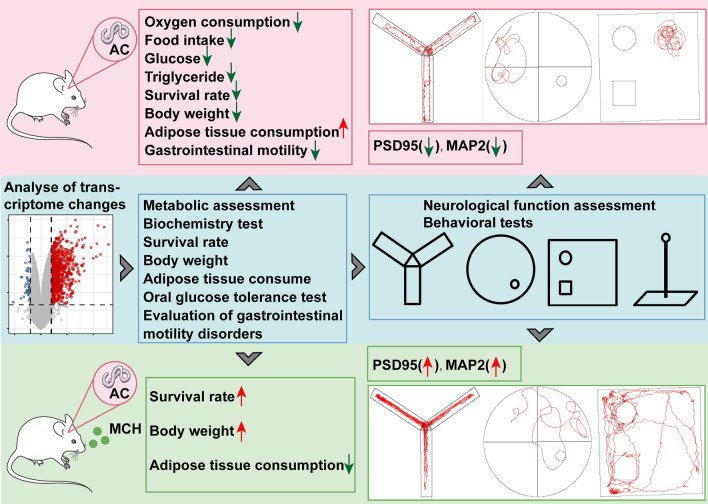

**Supplementary Information:**

The online version contains supplementary material available at 10.1186/s13071-024-06267-9.

## Background

*Angiostrongylus cantonensis* (AC), also known as rat lungworm, was first described by Chinese professor Hsin-Tao Chen in 1935 [1]. Rats are its primary hosts, while humans and mice are incidental hosts. AC infection typically occurs through the consumption of raw or undercooked snails, slugs, or contaminated food harboring infective larvae. In humans and mice, AC invasion into the brain can cause severe eosinophilic meningitis/encephalitis, characterized by increased eosinophils in the brain lining and symptoms such as headaches, neck stiffness, fever, nausea, vomiting, loss of appetite, and neurological manifestations such as coma, seizures, persistent sensory abnormalities, limb weakness, and even paralysis [2–4]. Untreated, this can result in long-term neurological damage. The main treatment for AC infection combines anthelmintic drugs and corticosteroids [5]; however, anthelmintics do not alleviate headaches [6, 7] and high-dose corticosteroids can have adverse effects [8]. Therefore, elucidating the pathogenic mechanisms of AC-induced neurofunctional damage and identifying effective neuroprotective drugs is crucial for improving patient prognosis.

In our previous studies, we discovered a significant link between neurofunctional impairment in AC-infected mice and the apoptosis and necrosis of astrocytes, neurons, and microglial cells in brain tissues [9, 10]. Analysis of brain gene expression microarrays from infected and control mice showed a notable upregulation of key chemokines, interleukins, and tumor necrosis factors post infection, with C–C motif chemokine ligand 8 (*Ccl8*) and chitinase-like 3 (*Chi3l3*) playing crucial roles in eosinophil recruitment and infiltration [11, 12]. The significant downregulation of pro-MCH (*Pmch*), Oxytocin (*Oxt*), and cocaine- and amphetamine-regulated transcript protein (*Cartpt*) post infection, all encoding neuropeptides, also garnered our attention. The roles of these neuropeptides in the progression of AC infection and the mechanisms through which AC suppresses their transcription levels remain largely unreported.

Melanin-concentrating hormone (MCH), a neuropeptide produced in hypothalamic neurons and encoded by the *Pmch* gene [13, 14], is pivotal in regulating various physiological functions. MCH neurons, projecting throughout the brain [15], are instrumental in managing processes including feeding, sleep, emotion, cognition, and movement. MCH operates primarily through melanin-concentrating hormone receptors 1 and 2 (MCHR1 and MCHR2), with MCHR1 being the sole receptor expressed in rodents [16]. Research in recent decades has extensively explored the role of MCH in regulating appetite, sleep, and energy expenditure [17, 18], spurring studies into MCHR1 as a potential target for obesity therapies [19, 20].

Recent discoveries indicate a significant association between MCH system abnormalities and certain central nervous system disorders, including Alzheimer’s disease (AD), Parkinson’s disease (PD), infectious encephalitis, and amyotrophic lateral sclerosis [21–24]. MCH administration in animal models is primarily conducted through intracerebroventricular injection and intranasal delivery, with the latter being a noninvasive route that circumvents the blood–brain barrier, enabling direct drug delivery from the nasal cavity to the brain. Postintranasal administration of MCH–Fluorescein Isothiocyanate (FITC) green fluorescence is observable in various brain regions, such as the olfactory bulb, frontal cortex, thalamus, hippocampus, and amygdala [25]. In AD mouse models induced by scopolamine and amyloid β-protein (Aβ), acute intranasal administration of MCH has shown to enhance memory retention, with long-term administration reducing soluble Aβ in the cortex of APP/PS1 double transgenic mice [26]. Additionally, MCH augments the phosphorylation of key proteins such as cAMP-response element binding protein (CREB), mitogen-activated protein kinase (MAPK), and glycogen synthase kinase-3β (GSK3β) in cortical and hippocampal tissues. Moreover, MCH has demonstrated analgesic effects in models of inflammatory and neuropathic pain, with its intranasal injection raising mechanical and thermal pain thresholds. These effects are reversible by naloxone and AM251, indicating the involvement of opioid and cannabinoid systems [27].

In consideration of preliminary findings indicating MCH anomalies during AC infection and given its observed roles in energy balance, neuroprotection, and analgesia in other models, this study aims to elucidate the role of MCH in AC infection development, understand the underlying mechanisms of its neuroprotective functions, and assess its potential as an adjunct therapeutic agent.

## Methods

### Animals and experimental design

Female BALB/c mice, aged 6–8 weeks and weighing 18–20 g, were obtained from Beijing Vital River Laboratory Animal Technology Co., Ltd. The mice were housed in a specific pathogen-free environment with controlled temperature, humidity, and a 12 h light/dark cycle. They had free access to sterilized water and standard diet. The experimental group details are presented in Additional file 1: Fig. S1.

### Infection with AC larvae

Third-stage larvae of *Biomphalaria glabrata*, maintained in our laboratory, were harvested following the protocol of a prior study [9]. Larvae counting was performed under a stereomicroscope (SZ650, Cnoptec). Each mouse in the infection group was orally administered 30 larvae.

### Drug administration

Intranasal application of MCH allows for direct brain access via the nasal–brain pathway [25]. MCH (070-47, Phoenix Biotech) was dissolved in sterile physiological saline, aliquoted, and stored at −80 °C until use. Mice began receiving MCH (1 μg/15 μL) on the day of infection and continued for 21 days. Control and AC group mice received an equivalent volume of sterile saline.

### Gene Ontology (GO) enrichment analysis

We retrieved differentially expressed genes between AC-infected mouse brain tissues and normal control groups from the Gene Expression Omnibus (GEO) database (GSE159486) [28]. These genes were visualized using volcano plots. The selected genes, based on the criterion of |log2(fold change)|> 1, underwent GO enrichment analysis using the Hiplot Pro online platform [29] (https://hiplot.com.cn/).

### Real‑time quantitative PCR (RT–qPCR)

Total RNA was extracted from the hypothalamus and adipose tissue using RNAex Pro RNA extraction reagent (AG21102, AG). RNA concentration and purity were assessed using a NanoDrop One spectrophotometer. Reverse transcription to cDNA was performed using Evo M-MLV Reverse Transcription Premix (AG11706, AG). RT–qPCR was conducted on a LightCycler 480 II instrument, using a 10 μL reaction mixture that included specific gene primers (detailed in Additional file 5: Table S1). *Actb* was the reference gene. Amplification involved an initial denaturation at 95 °C for 30 s, followed by 40 cycles of 95 °C for 5 s, and 60 °C for 30 s, with fluorescence data collection after each cycle. Relative gene expression was quantified using the 2^−ΔΔCt^ method.

### Immunofluorescence

Mice were anesthetized deeply using isoflurane (R510-22, RWD Life Science) and subjected to transcardial perfusion with physiological saline followed by 4% paraformaldehyde (G1101, Servicebio) prior to brain extraction. Brains were postfixed in 4% paraformaldehyde overnight, sequentially dehydrated in 20% sucrose solution for 24 h, and then in 30% sucrose solution for 48 h. The brains were subsequently embedded in optimal cutting temperature compound (G6059, Servicebio) and sectioned at 16 μm thickness using a cryostat (NX50, Thermo Fisher Scientific). Clean slides (G6012-2, Servicebio) were used to mount the tissue sections, which were then stored at −20 °C.

The frozen sections were warmed at 37 °C for 10–20 min to remove moisture and refixed in 4% paraformaldehyde for 30 min. Following three washes with phosphate-buffered saline (PBS), antigen retrieval solution was applied. After three additional PBS washes, immunofluorescence permeabilization solution was added, and the sections incubated for 10 min. A 3% bovine serum albumin solution was applied for 30 min incubation. Sections were then incubated overnight at 4 °C with the following primary antibodies as needed: MCH (1:3000, H-070-47, Phoenix Biotech), neuronal nuclei (NeuN) (1:500, 94403, Cell signaling technology), glial fibrillary acidic protein (GFAP) (1:500, 3670, Cell signaling technology). After 14 h, the sections were washed three times in PBS. The sections were then incubated for 50 min at room temperature in the dark with the following secondary antibodies as appropriate: Alexa Flour 488-conjugated goat anti-rabbit IgG (1:500, ab150077, Abcam), Alexa Flour 488-conjugated goat anti-mouse IgG (1:500, ab150113, Abcam), Alexa Flour 594-conjugated goat anti-rabbit IgG (1:500, ab150080, Abcam). Following this, the sections were washed three times with PBS. Then 4′,6-diamidino-2-phenylindol (DAPI) staining solution was added, and the sections were incubated for 10 min at room temperature in the dark. Post staining, images were captured using a digital slide scanner (KF-FL-400, Ningbo Konfoong Bioinformation Tech) or a laser scanning confocal microscope (Nikon C2, Nikon).

### Histopathology and immunohistochemistry

Isolated brown adipose tissue (BAT), inguinal white adipose tissue (iWAT), and gonadal white adipose tissue (gWAT) were fixed in a specialized fat-fixing solution (G1119, Servicebio) for 24 h. Tissue blocks underwent sequential dehydration, clearing, and impregnation with paraffin, followed by embedding in paraffin wax, using ethanol, xylene, and paraffin. Prepared wax blocks were sectioned at 4 μm using a paraffin microtome (RM2235, Leica). Before usage, sections were placed in a 60 °C oven for 30 min. Deparaffinization of tissue sections was performed in xylene, followed by dehydration through a graded alcohol series. Some sections were stained following the hematoxylin and eosin (H&E) staining kit (G1076, Servicebio) protocol. Adipocyte number and volume were observed under a brightfield microscope (BX63, Olympus) and quantified using ImageJ software. Another set of sections was used for immunohistochemistry to detect mitochondrial brown fat uncoupling protein 1 (UCP1) expression in adipose tissues. Sections were treated with 0.3% hydrogen peroxide for 10 min to block endogenous enzyme activity, followed by overnight incubation at 4 °C with UCP1-specific antibody (1:1000, ab209483, Abcam). HRP-conjugated goat anti-rabbit IgG (1:2000, AB0101, Abways) was added for 1 h incubation at 37 °C. Diaminobenzidine (G1212, Servicebio) was used as the chromogen. Images were captured with a digital slide scanner–brightfield (KF-PRO-120-H1, Ningbo Konfoong Bioinformation Tech).

### Metabolic assessment

Mice were placed in a metabolic indirect calorimetry system (Sable Promethion CORE, Sable Systems International) to measure oxygen consumption, carbon dioxide production, food and water intake, and wheel running activity. The mice were kept under a 12 h light/dark cycle at 24 °C with free access to food and water. Metabolic parameters were measured using *XY* laser beam arrays to monitor movement. The initial 24 h were considered an adaptation period and excluded from data analysis. Data were analyzed and visualized using the online platform CalR [30] (https://CalRapp.org/).

### Biochemistry test

Mouse serum levels of glucose (GLU), triglycerides (TG), low-density lipoprotein (LDL), high-density lipoprotein (HDL), and total cholesterol (TC) were determined using a biochemical analyzer (3100, Hitachi).

### Oral GLU tolerance test

Mice underwent a 16 h fasting period, with access to water, prior to testing. Body weights were recorded before administering a 50% GLU solution (2 g/kg) via oral gavage. The tail tip (0.3 cm) was snipped using sterilized scissors, and the first blood drop was discarded. Blood GLU levels were measured at 0, 15, 30, 60, 90, and 120 min using a Roche blood GLU meter and corresponding test strips (ACCU-CHEK Guide, Roche).

### Evaluation of gastrointestinal motility disorders

Mice were fasted for 16 h with access to water before testing. One group received a 200 μL gavage of a suspension containing sodium carboxymethylcellulose, milk powder, sucrose, and Evans blue. The time to the first colored stool passage and total feces amount over 8 h were recorded. Another group received the same gavage, and after 30 min, they were anesthetized with isoflurane and euthanized. The abdomen was dissected, exposing the intestine. The intestine was ligated at the lower end of the pylorus and ileocecal junction and then cut. The mesentery was carefully dissected along the intestine, and the small intestine was isolated and laid out without stretching. The total length of the small intestine and the distance from the front end of the suspension to the pylorus were measured to calculate the gastrointestinal transit index using the formula: gastrointestinal transit index (%) = (distance from the front end of the suspension to the pylorus / total length of the small intestine) × 100%.

### Survival rate, body weight, and neurological function assessment

Daily monitoring of mouse survival and body weight commenced from the first day of infection. Neurological function was evaluated using a scoring system as described in previous studies [31]. Briefly, each observed change was assigned a score. Motor scoring (0–12) comprised assessments of voluntary movement within 5 min, limb symmetry, climbing ability, and balance. Sensory scoring (5–15) involved evaluating proprioception, tactile, visual, olfactory, and nociceptive responses. Each aspect was scored separately, accumulating to a maximum total of 27.

### Behavioral tests

Motor coordination and cognitive function were assessed through a Y maze, a Morris water maze (MWM), new object recognition (NOR) tests, and a pole test. Trajectory, distance, and speed data were captured using an integrated behavioral recognition analysis system (PhenoScan, Clever Sys. Inc.). Mice were acclimatized to the environment for 1 h before commencing behavioral experiments. In the Y-maze test, mice were placed in the maze’s center, allowed 10 min of continuous autonomous alternation, and their arm entry sequence was recorded. The autonomous alternation rate (%) was calculated as the number of alternations divided by (total entries, 2) %. This rate indicates the extent of spontaneous, continuous alternation choice, with higher percentages reflecting better working memory. MWM testing included a visible platform phase on day 1 and a hidden platform phase on days 2–4. Mice were placed in the pool four times daily, once in each quadrant. Animals locating the platform rested for 20 s; those failing within 70 s were guided to the platform for a similar rest period. On day 5, a platform-free phase, mice were released in the third quadrant, and metrics such as time spent in the target quadrant, platform crossing count, average swimming distance, and speed were recorded. For the NOR test, mice were initially placed in a 40 cm^3^ test box for a 10 min exploration on day 1. On day 2, two identical objects were introduced for a 10 min exploration. On day 3, one object was replaced, and the mice explored for 5 min. Recognition index (%) = (time spent on new object) / (time spent on new object + time spent on old object) × 100%. The pole test apparatus consisted of a wooden pole (50 cm high, 1 cm in diameter, wrapped with gauze) with a ball at the top. Mice were pretrained with the pole three times to make sure that they would turn head down once were put on the ball. During the pole test, the total time it took for the mouse to get from the top to the bottom was measured.

### Western blotting

The expression of synapse-related proteins was measured by western blotting, such as postsynaptic density protein 95 (PSD95), microtubule-associated protein-2 (MAP2), and synaptophysin (SYP). Cortex separated from the brain were homogenized in a RIPA lysis buffer (89900, Thermo Fisher Scientific) containing protease and phosphatase inhibitor cocktail (P1050, Beyotime) and quantified with a BCA protein assay kit (P0010, Beyotime). Then, 37.5 μg protein was electrophoresed on 10% sodium dodecyl sulfate–polyacrylamide gel electrophoresis and transferred to polyvinylidene fluoride membranes with 110 V for 2 h. The membranes were incubated with 3% nonfat milk at room temperature for 1 h. Then, the membranes incubated with the following primary antibodies overnight at 4 °C: anti-MAP2 (1:1000, 8707, Cell Signaling Technology), anti-PSD95(1:1000, ab2723, Abcam), anti-SYP (1:500, ab8049, Abcam), and anti-β-actin (3700, Cell Signaling Technology). After washing three times with tris-buffered saline and Tween 20, the membranes were incubated with secondary antibodies at room temperature for 1 h: Goat Anti-Rabbit IgG (H + L) HRP (1:10,000, AB0101, Abways) and Goat Anti-Mouse IgG (H + L) HRP (1:10,000, AB0102, Abways). The protein bands were visualized using a super-sensitive chemiluminescence reagent (180–506, Tanon) on a chemiluminescence imaging system (Gelview 6000Plus, Biolight). The density of the protein bands was measured using ImageJ software.

### Statistical analysis

Data were presented as mean ± standard deviation (SD). GraphPad Prism was utilized for statistical analysis. The unpaired two-tailed *t*-test was applied to assess differences between two groups. One-way analysis of variance (ANOVA) was employed for multi-group comparisons. A *P*-value < 0.05 was considered to indicate statistical significance.

## Results

### Decrease in MCH expression observed in the AC-infected mouse model

**Fig. 1:**
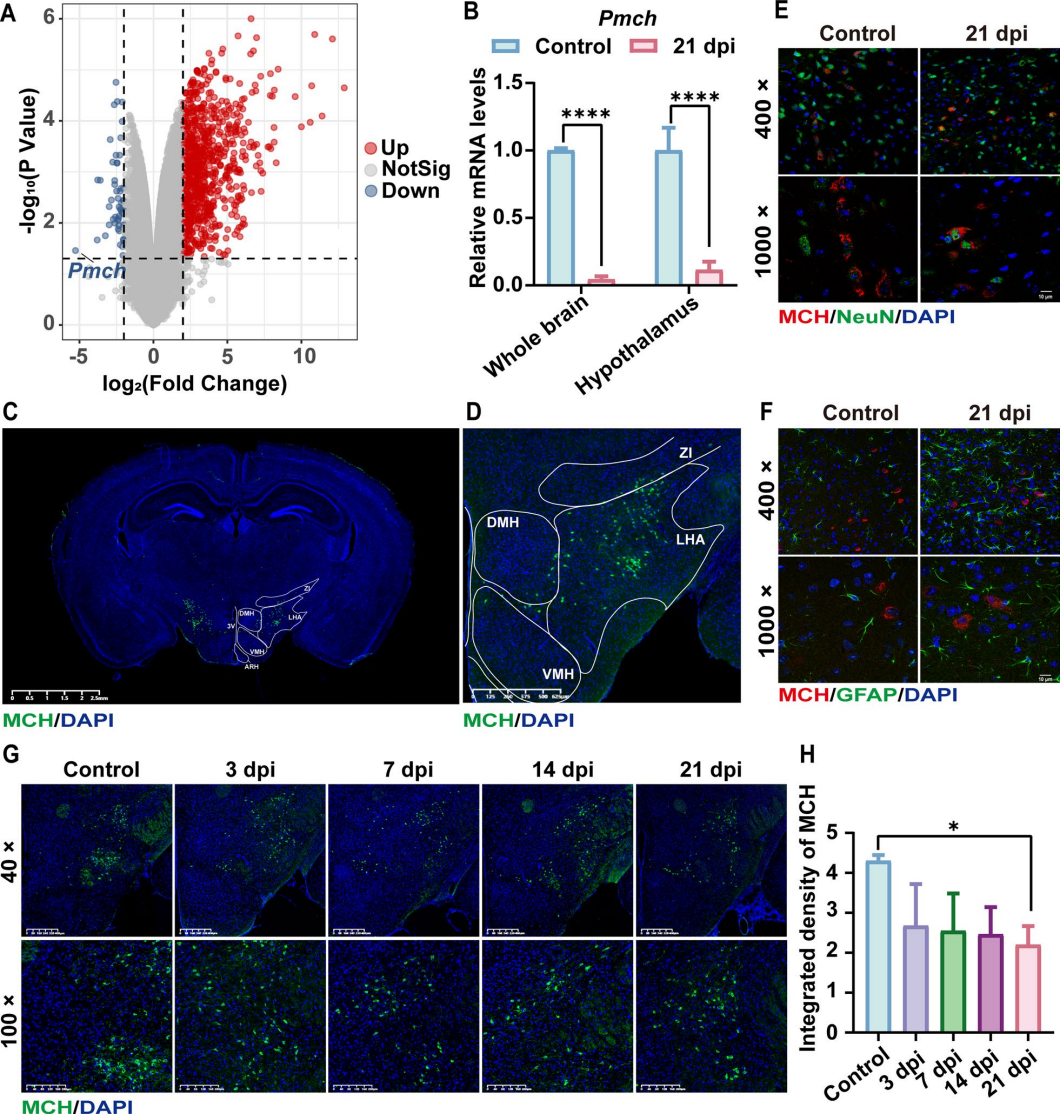
AC infection reduces MCH expression in mice. **A** Differential volcano plot illustrating brain transcriptome changes in AC-infected mice. **B** RT–qPCR analysis of *Pmch* mRNA transcription levels in the whole brain (*n* = 3) and hypothalamus (*n* = 4). **C**, **D** Panoramic localization of MCH in coronal brain sections. Part **D** is an enlarged view of part **C**. **E**, **F** Representative images of MCH costained with NeuN and GFAP. **G**, **H** Representative images depicting MCH immunofluorescence in hypothalamic regions, along with fluorescence intensity statistics (*n* = 3). Data are presented as mean ± SD. Compared with the control group, statistical significance is denoted as *P < 0.05, ****P < 0.0001. *NotSig* not significant, *Pmch* pro-melanin-concentrating hormone, *dpi* days post infection, *MCH* melanin-concentrating hormone, *DAPI* 4′,6-diamidino-2-phenylindole, *NeuN* neuronal nuclei, *GFAP* glial fibrillary acidic protein, *3V* 3rd ventricle, *ARH* hypothalamic arcuate nucleus, *DMH* dorsomedial hypothalamus, *LHA* lateral hypothalamic area, *VMH* ventromedial hypothalamic nucleus, *ZI* zona incerta

Transcriptome data visualization indicated a significant downregulation of *Pmch* in the infected group (Fig. 1A). Genes that were up- and downregulated underwent GO enrichment analysis. Upregulated genes predominantly pertained to biological processes such as regulation of immune effector processes, cytokine-mediated signaling pathways, and regulation of inflammatory responses. Notably, downregulated genes were enriched in processes such as feeding behavior, learning and memory, and neuropeptide signaling pathways (Additional file 2: Fig. S2). Subsequently, RT–qPCR analysis of the whole brain (*P* < 0.0001) and hypothalamic tissues (*P* < 0.0001) confirmed a substantial reduction in *Pmch* transcript levels at 21 days post infection (dpi) (Fig. 1B). Additionally, immunofluorescence was employed to ascertain the brain distribution and localization of MCH (Fig. 1C, D). The findings indicated that MCH predominantly localized in neurons in the hypothalamic region, aligning with previous reports [14], rather than in astrocytes or other regions (Fig. 1E, F). Moreover, at 21 dpi, the fluorescence intensity of MCH in the hypothalamus was significantly lower compared with the control group (*P* < 0.05) (Fig. 1G, H). Although a decreasing trend was observed at 3, 7, and 14 dpi, the differences were not statistically significant.

### AC infection induces energy metabolism imbalance in mice

**Fig. 2:**
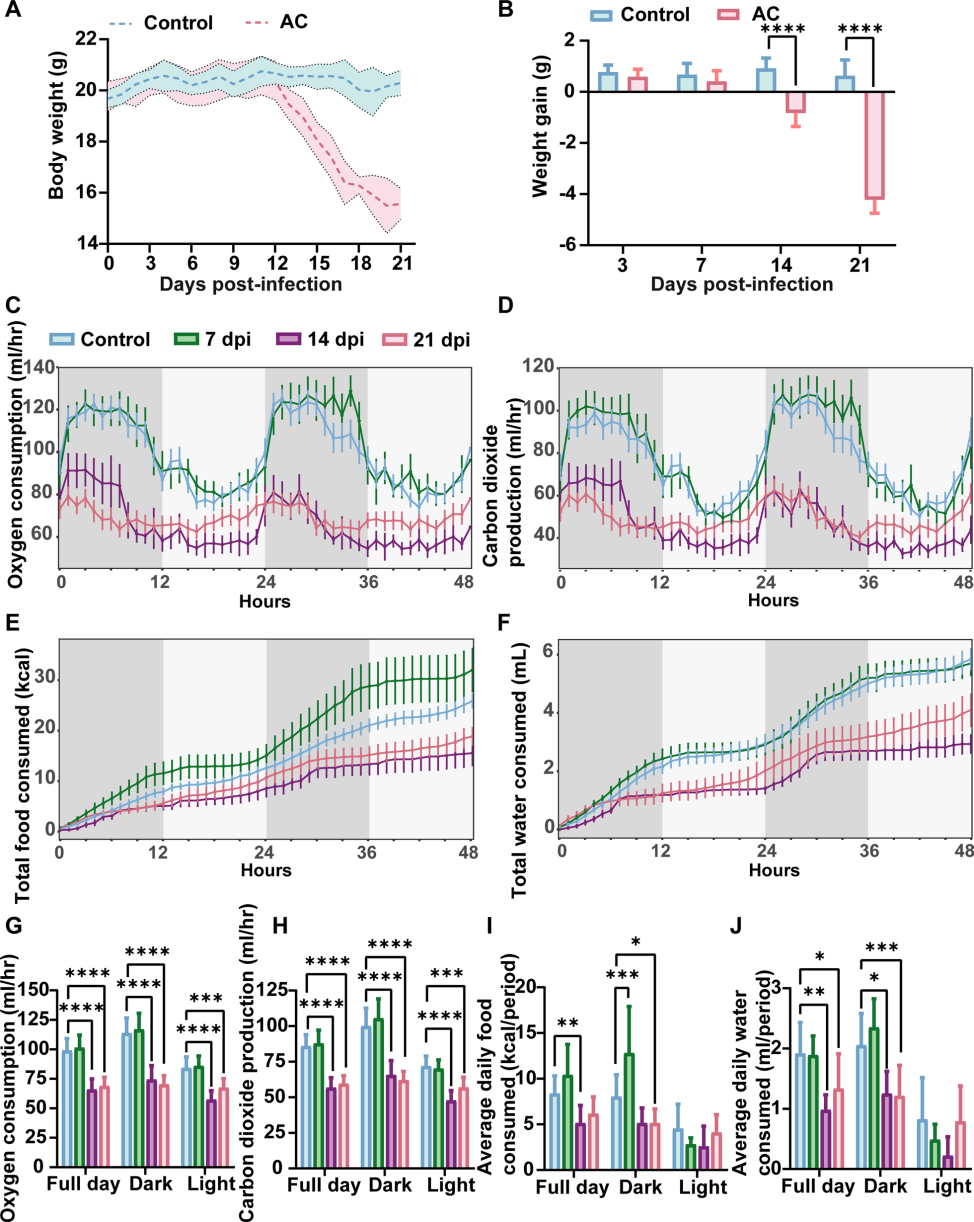
AC Infection causes energy metabolism imbalance in mice. **A** Mouse body weight change curve. **B** Weight increment statistics at different infection stages. **C**, **G** Oxygen consumption curve and statistics in mice within 48 h. **D**, **H** Carbon dioxide production curve and statistics in mice within 48 h. **E**, **I** Food consumed curve and statistics in mice within 48 h. **F**, **J** Water consumed curve and statistics in mice within 48 h. Data are presented as mean ± SD, *n* ≥ 5. Compared with the control group, statistical significance is denoted as *P < 0.05, **P < 0.01, ***P < 0.001, ****P < 0.0001. *AC* Angiostrongylus cantonensis, *dpi* days post infection

Given the established role of MCH in regulating energy metabolism, cognition, and motor processes, we investigated these aspects in the AC model. In the control group, mice displayed normal behavior, coat condition, diet, and defecation, with stable body weight. In contrast, the infected mice exhibited lethargy, coarse coats, and significant weight loss. Weight reduction became evident from 13 dpi (Fig. 2A), with marked decreases noted at 14 and 21 dpi (*P* < 0.0001) (Fig. 2B). We monitored oxygen consumption, carbon dioxide production, consumed of food and water in control and infected mice at various postinfection intervals. While no significant changes were observed for most of the measures at 7 dpi, notable decreases in these parameters were recorded at 14 and 21 dpi compared with controls (Fig. 2C–J). Gastrointestinal motility assessment revealed prolonged expulsion times for the first stained feces (*P* < 0.05), increased gastric residual rate (*P* < 0.01), and significant reductions in fecal pellet frequency, fecal pellet output and intestinal propulsion rate (*P* < 0.05) in the infected group, with no notable difference in fecal moisture content, indicating gastrointestinal motility disorders post-AC infection (Additional file 3: Fig. S3).

### AC infection leads to extensive adipose tissue loss in mice

**Fig. 3:**
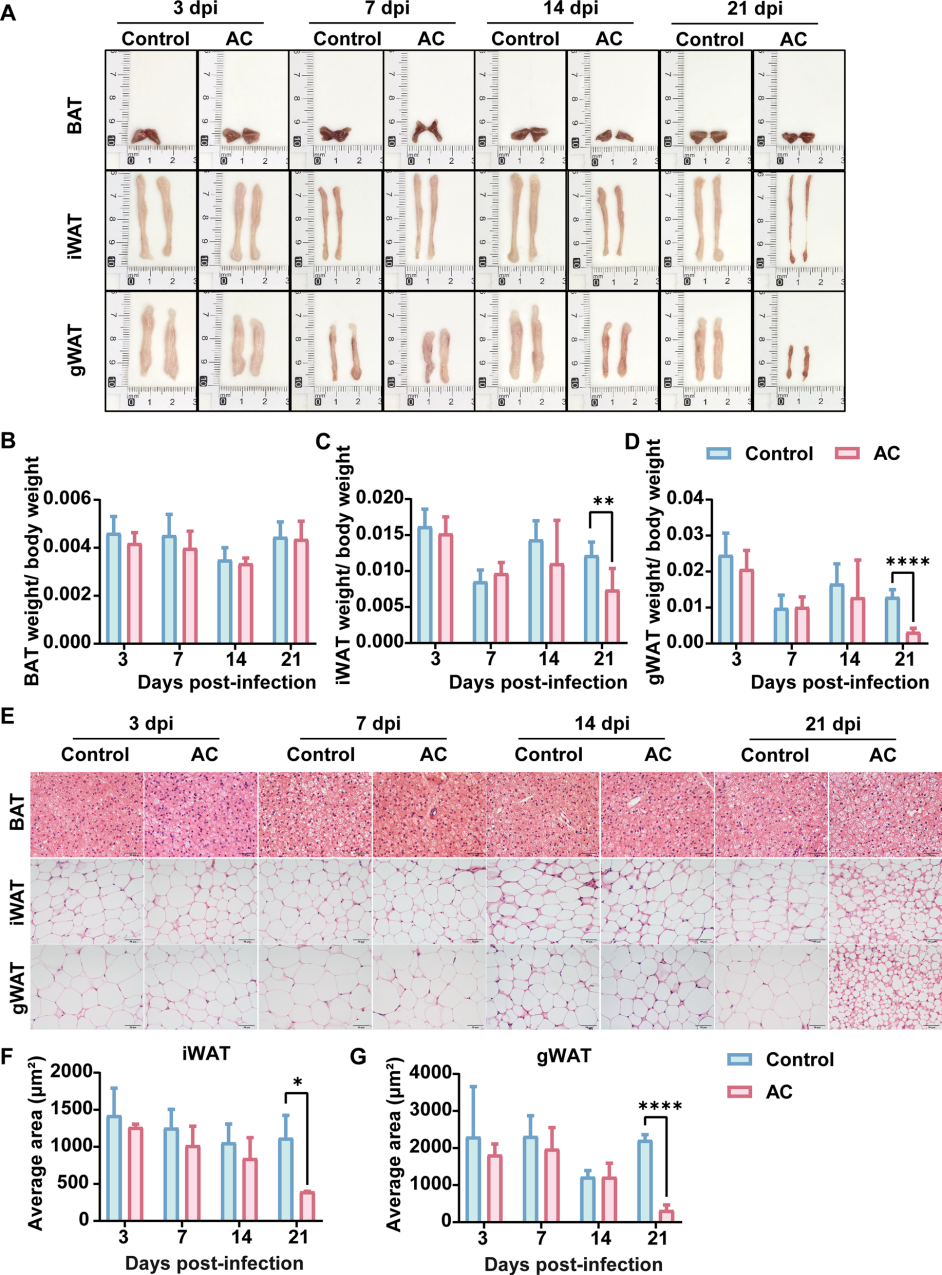
AC infection leads to extensive loss of adipose tissue in mice. **A** Macroscopic images. **B**, **C**, **D** Statistical chart of tissue weight proportion to body weight in different parts (*n* = 6). **E** Pathological sections of adipose tissue. **F**, **G** Statistical chart of the average single-cell area of iWAT and gWAT (*n* = 3). Data are presented as mean ± SD. Compared with the control group, statistical significance is denoted as *P < 0.05, **P < 0.01, ****P < 0.0001. *BAT* brown adipose tissue, *iWAT* inguinal white adipose tissue, *gWAT* gonadal white adipose tissue

We meticulously separated, photographed, and weighed BAT, iWAT, and gWAT from control and infected mice. Histological examinations with H&E staining were conducted to assess pathological changes in adipose tissue post-AC infection. Visual and quantitative evaluations revealed significant reductions in white adipose tissue volume at 14 and 21 dpi (Fig. 3A). Notably, both iWAT and gWAT showed marked reductions at 21 dpi (*P* < 0.01 and *P* < 0.0001, respectively), indicative of extensive fat breakdown in response to infection (Fig. 3B–D). At 21 dpi, H&E staining and quantitative analysis demonstrated significantly smaller average cell areas in iWAT (*P* < 0.05) and gWAT (*P* < 0.0001) in infected mice compared with controls (Fig. 3E–G), with no significant differences observed at 3, 7, and 14 dpi.

### Metabolic imbalance in GLU and lipid metabolism due to AC infection in mice

**Fig. 4:**
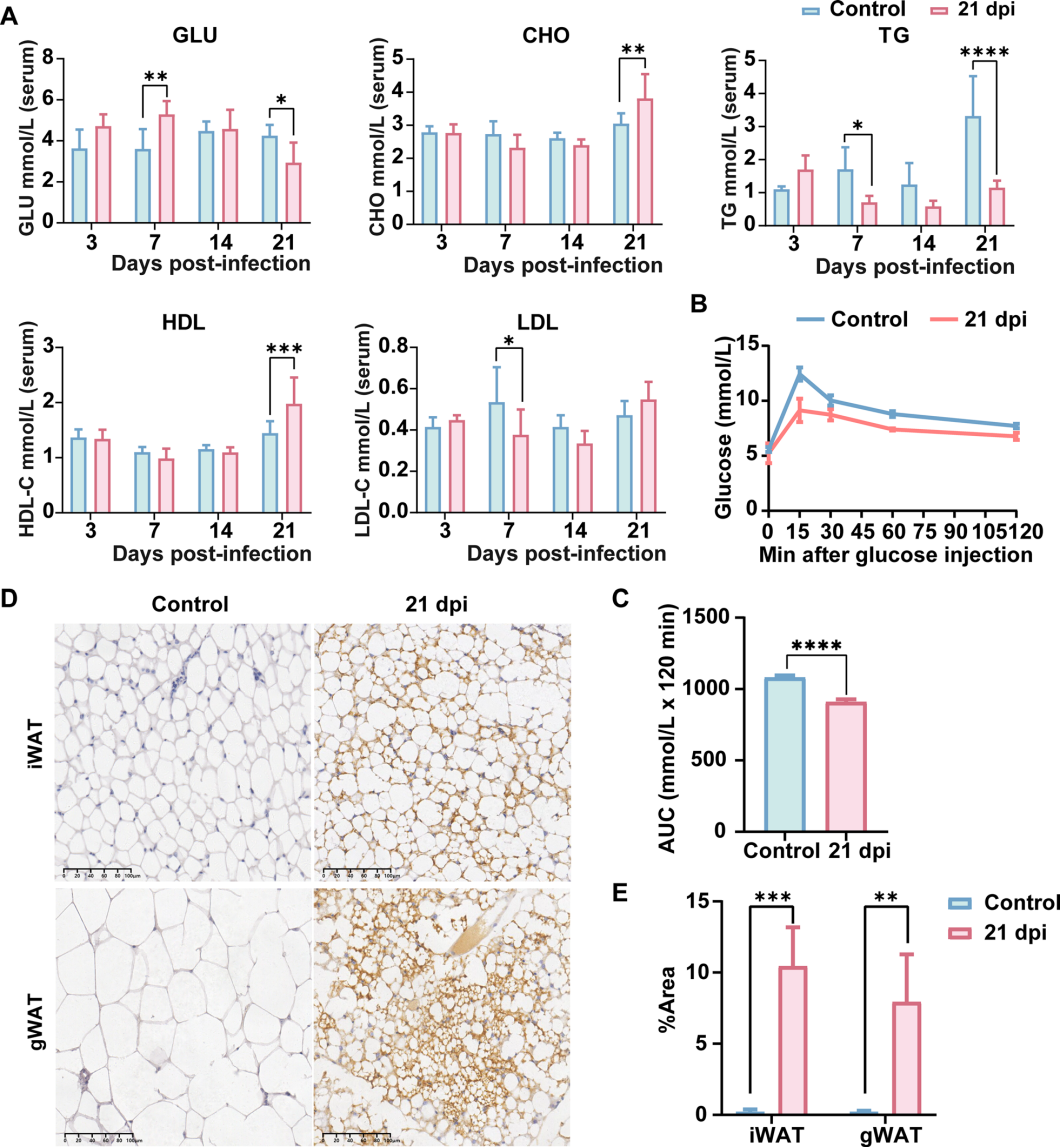
AC infection causes imbalance in GLU and lipid metabolism in mice. **A** Serum biochemical tests (*n* = 6), including GLU, CHO, TG, HDL, LDL. **B**, **C** GLU tolerance and AUC curve (*n* = 3). **D**, **E** Immunohistochemical images and statistics of UCP1 in mouse gWAT (*n* = 3). Data are presented as mean ± SD. Compared with the control group, statistical significance is indicated as *P < 0.05, **P < 0.01, ***P < 0.001, ****P < 0.0001. *GLU* glucose, *TG* triglycerides, *LDL* low-density lipoprotein, *HDL* high-density lipoprotein, *TC* total cholesterol, *AUC* area under the curve

To elucidate the alterations in GLU and lipid metabolism in peripheral circulation of mice, serum samples were analyzed biochemically. The results, as depicted in Fig. 4A, showed significant reductions in GLU (*P* < 0.05) and TG (*P* < 0.0001) levels at 21 dpi in infected mice, indicative of substantial GLU and fat consumption. Concurrently, increases in CHO (*P* < 0.01) and HDL (*P* < 0.001) were observed, potentially implicating altered cholesterol metabolism. The GLU tolerance curve demonstrated a higher GLU clearance rate in the infected mice compared with controls (*P* < 0.0001) (Fig. 4B, C). Further investigations into adipose tissue changes during AC infection included examining transcription levels of lipid metabolism enzymes and thermogenesis-related genes in gWAT (Additional file 4: Fig. S4), along with in situ expression of UCP1. At 21 dpi, the fatty acid synthase gene (*Fasn*) showed a significant decrease (*P* < 0.05), whereas genes related to fatty acid catabolism, such as Patatin-like phospholipase domain-containing protein 2 (*Atgl*) (*P* < 0.01) and lysosomal acid lipase A (*Lipa*) (*P* < 0.0001), and the fatty acid β-oxidation gene carnitine palmitoyltransferase 1a, liver (*Cpt1α*) (*P* < 0.01), exhibited significant increases. Additionally, the gene associated with browning and heat production, peroxisome proliferator-activated receptor gamma coactivator 1-alpha (*Pgc1α*), was significantly upregulated (*P* < 0.01). Immunohistochemical analysis revealed heightened expression of UCP1 in iWAT (*P* < 0.001) and gWAT (*P* < 0.01) of the infected group (Fig. 4D, E). These findings suggest that post-AC infection, mice counteract the infection through extensive breakdown of adipose tissue, reduced fat synthesis, accelerated β-oxidation, and increased thermogenesis for energy provision.

### Neurological impairment and dyskinesia in mice due to AC infection

**Fig. 5:**
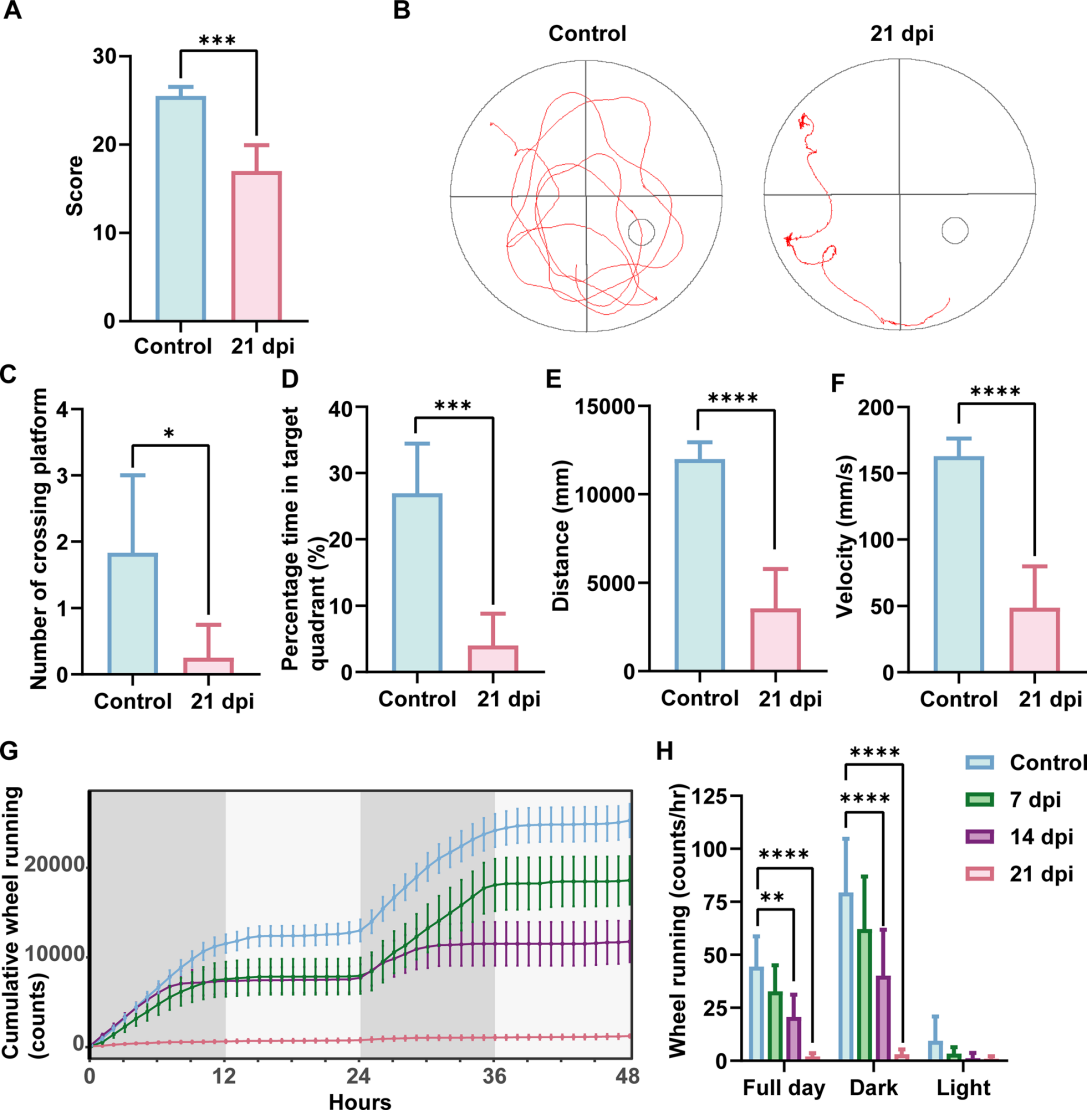
AC infection causes neurological impairment and dyskinesia in mice. **A** Neurological function score (*n* ≥ 4). **B** MWM trajectory. **C**, **D**, **E**, **F** Statistics of platform crossings, percentage time in target quadrant, distance, and velocity in MWM (*n* ≥ 4). **G**, **H** Changes in running wheel activity and statistics (*n* ≥ 5). Data are presented as mean ± SD. Compared with the control group, statistical significance is indicated as *P < 0.05, **P < 0.01, ***P < 0.001, ****P < 0.0001. *dpi* days post infection

Considering AC’s primary impact on the central nervous system in mice, neurological functions were assessed using scoring methods and MWM. Additionally, an energy metabolism assay system recorded the mice’s running wheel activity. At 21 dpi, the infected mice demonstrated significant neurological impairment (*P* < 0.001) (Fig. 5A), characterized by a lack of back-and-forth movement, inability to grasp the cage lid, and failure to remain atop a 2 cm diameter column. MWM revealed that infected mice struggled to locate the platform (Fig. 5B). Statistical analysis showed significant reductions in platform crossings (*P* < 0.05), percentage time in the target quadrant (*P* < 0.001), movement distance (*P* < 0.0001), and velocity (*P* < 0.0001) compared with the control group (Fig. 5C–F). Moreover, running wheel exercise data indicated a progressive decline in locomotor ability in infected mice, culminating in minimal participation at 21 dpi (*P* < 0.0001) (Fig. 5G, H).

### MCH enhances survival, body, and adipose tissue weight in AC-infected mice

**Fig. 6:**
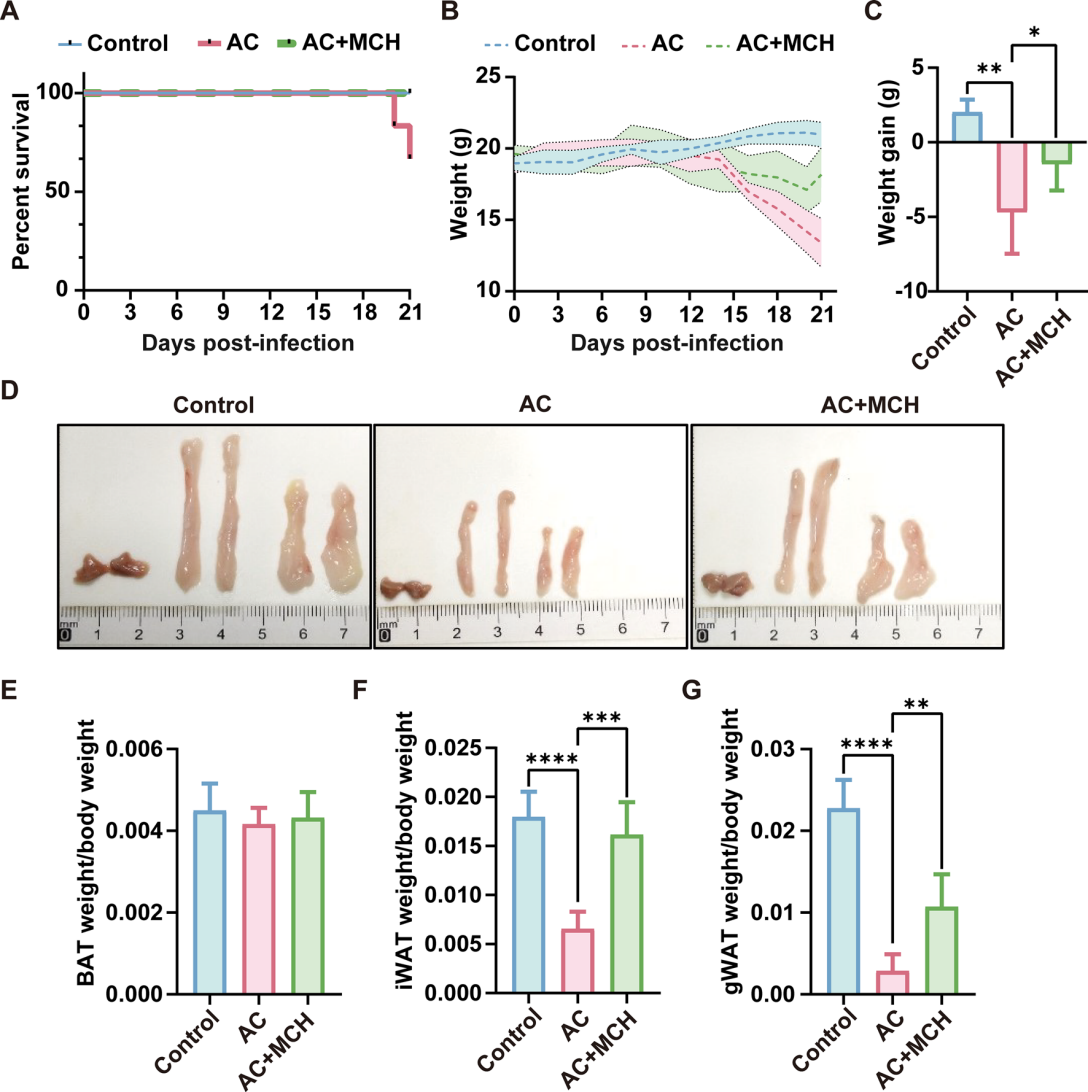
MCH improves survival, body weight, and adipose tissue weight in mice. **A** Survival curve (*n* = 6). **B** Mouse body weight change curve (*n* ≥ 4). **C** Weight increment in mice (*n* ≥ 4). **D** Macroscopic images of adipose tissue. **E**, **F**, **G** Statistical chart of adipose tissue weight proportion to body weight (*n* ≥ 4). Data are presented as mean ± SD. Compared with the AC group, statistical significance is indicated as *P < 0.05, **P < 0.01, ***P < 0.001, ****P < 0.0001. *AC* Angiostrongylus cantonensis, *MCH* melanin-concentrating hormone, *BAT* brown adipose tissue, *iWAT* inguinal white adipose tissue, *gWAT* gonadal white adipose tissue

Postadministration of MCH via nasal drip, there was a notable improvement in the general demeanor and fur condition of the mice. Mice in the AC + MCH group exhibited extended survival times compared with the AC-only group (Fig. 6A). Additionally, as depicted in Fig. 6B and C, weight loss associated with AC infection was mitigated in the AC + MCH group (*P* < 0.05). Observation and weighing of adipose tissues revealed that the weights of iWAT (*P* < 0.001) and gWAT (*P* < 0.01) were significantly higher in the AC + MCH group compared with the AC group (Fig. 6D, E).

### MCH mitigates dyskinesia in mice

**Fig. 7:**
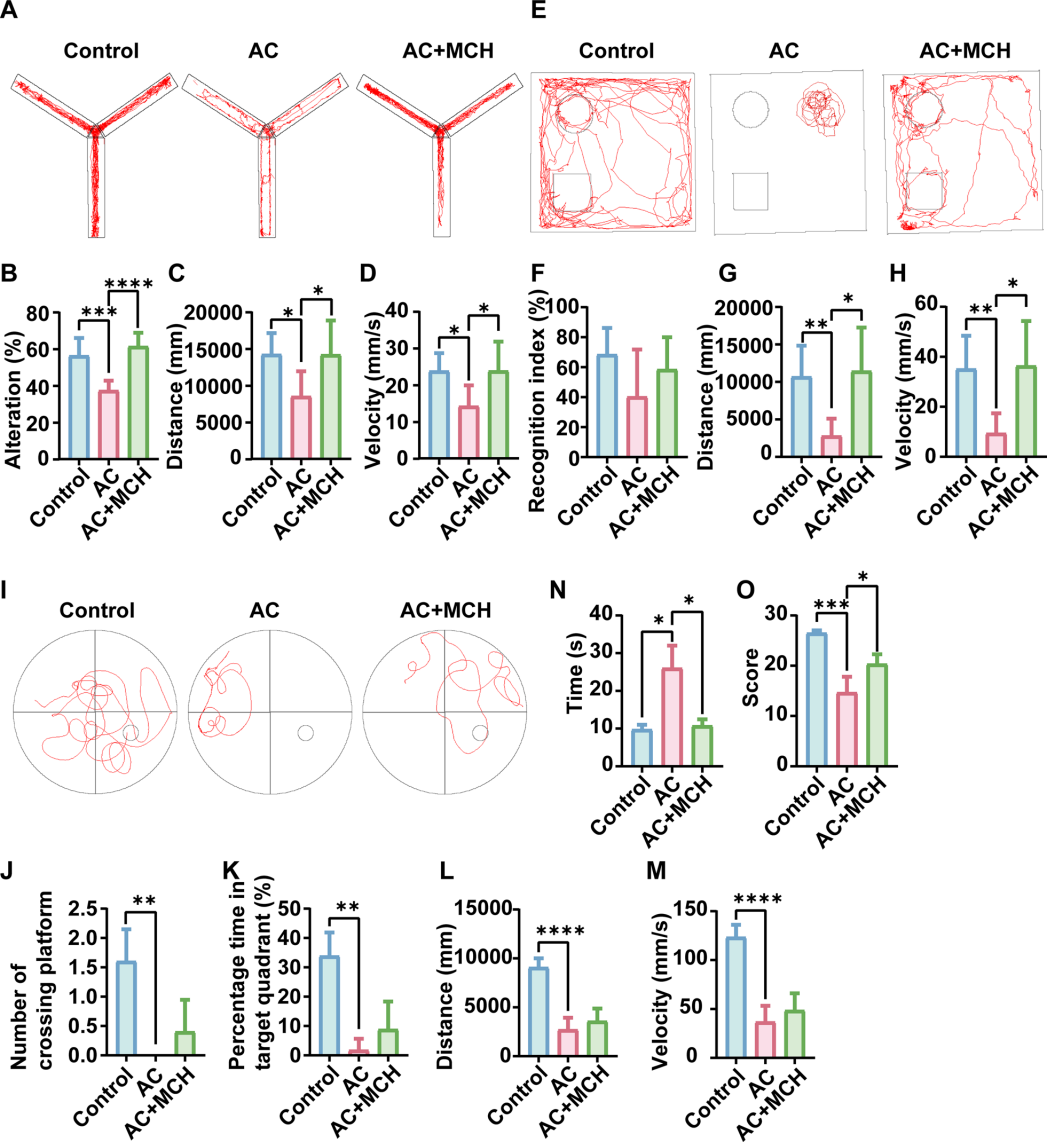
MCH improves neurological function and dyskinesia in mice. **A** Y-maze movement trajectory. **B**, **C**, **D** Statistics of Y-maze free alternation rate, distance, and velocity (*n* ≥ 6). **E** NOR test trajectory. **F**, **G**, **H** Statistics of recognition index, mouse travel distance, and velocity in NOR test (*n* ≥ 5). **I** MWM movement trajectory. **J**, **K**, **L**, **M** Statistics of platform crossings, percentage time in target quadrant, distance, and velocity in MWM (*n* ≥ 5). **N** Statistics of the time spent in the pole test (*n* = 4). **O** Neurological function score (*n* = 6). Data are presented as mean ± SD. Compared with the AC group, statistical significance is indicated as *P < 0.05, **P < 0.01, ***P < 0.001, ****P < 0.0001. *AC* Angiostrongylus cantonensis, *MCH* melanin-concentrating hormone

Cognitive and locomotor functions in mice were evaluated using neurological function scores, Y maze, MWM, NOR, and pole test. In the Y maze, the AC group showed a reduced free alternation rate (*P* < 0.001), with shorter distances and slower velocity (Fig. 7A–D). However, MCH administration improved these metrics. Figure 7E–H demonstrates limited movement and object exploration in NOR. Post-MCH administration, there was a significant improvement in movement distance and speed (*P* < 0.05). During the MWM no-platform phase, there is a trend that MCH treatment led to an increase in the number of platform crossings and time spent in the target quadrant (Fig. 7I–M). In the pole test, the control group and AC + MCH group were able to climb to the bottom of the pole in a shorter time (*P* < 0.05) (Fig. 7N). However, the AC group even fell directly. In the AC + MCH group, improvements were noted in locomotion, climbing, and balance abilities, as reflected in the substantial increase in neurological function scores (*P* < 0.05) (Fig. 7O).

### MCH rescues AC-induced changes of PSD95, MAP2, and BCL2

**Fig. 8:**
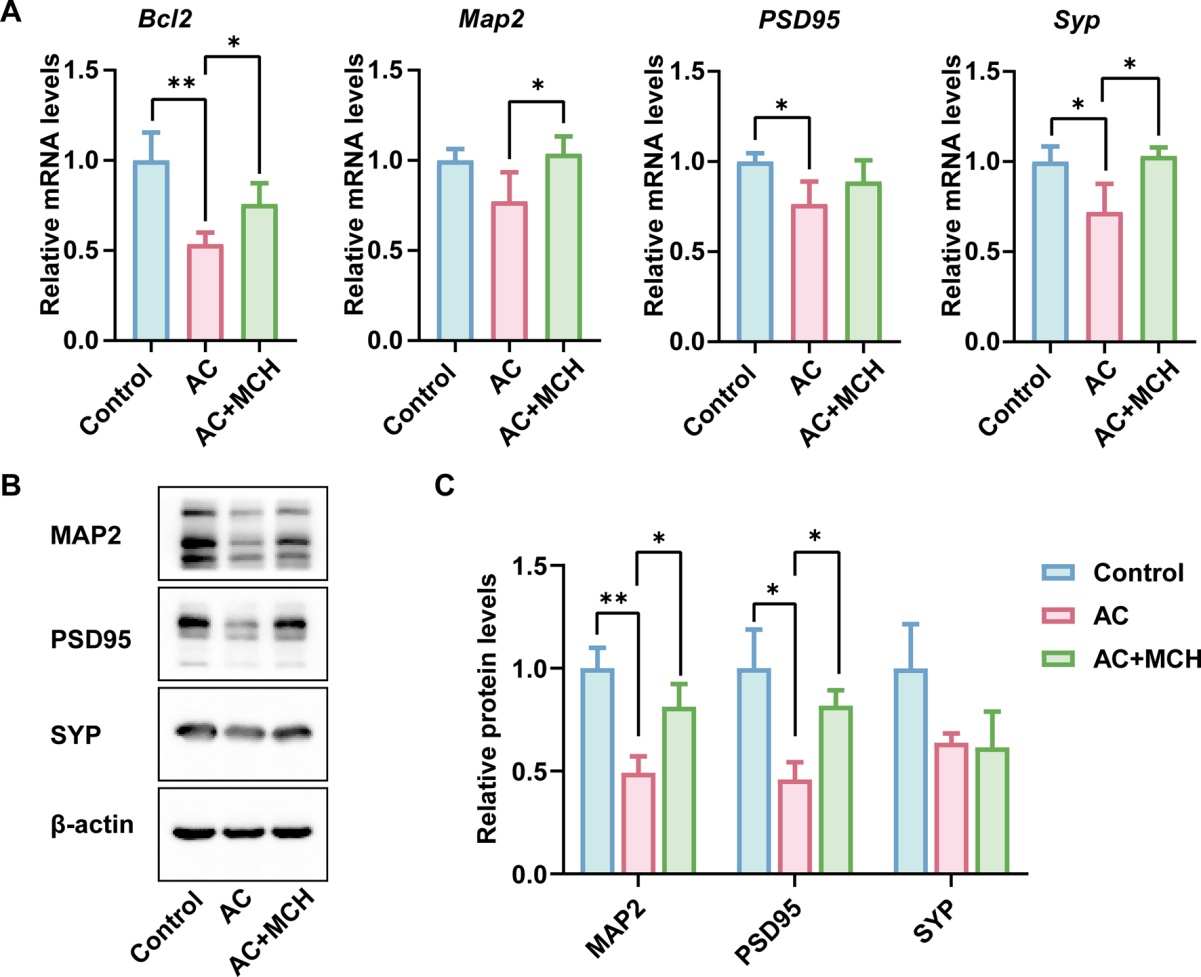
Effect of intranasal MCH on synapse-related proteins. **A** Transcription levels of *Bcl2*, *Map2*, *PSD95*, *Syp* in mouse cortex (*n* = 5). **B** The expression levels of MAP2, PSD95, and SYP were evaluated by western blotting. **C** Densitometrical quantification of the blots after normalizing with β-actin (*n* = 3). Data are presented as mean ± SD. Compared with the AC group, statistical significance is indicated as *P < 0.05, **P < 0.01. *Bcl2* B cell leukemia/lymphoma 2, *Map2* microtubule-associated protein 2, *PSD95* postsynaptic density protein 95, *Syp* Synaptophysin, *AC* Angiostrongylus cantonensis, *MCH* melanin-concentrating hormone

The transmission of nerve signals relies on synapses, which in turn depend on the normal expression of synapse-related proteins such as PSD95 and SYP. To investigate how MCH improves dyskinesia induced by AC infection, we conducted RT–qPCR and western blotting to examine the levels of synapse-related proteins in cortex. Our findings indicate that infection significantly reduces PSD95 expression (*P* < 0.05), while MCH treatment fully restores its relative strength (*P* < 0.05) (Fig. 8B, C). Although there were significant changes in *Syp* at the transcriptional level (*P* < 0.05) (Fig. 8A), there were not statistically significant at the protein level. MAP2 is a crucial component of neuron cytoskeleton; our results show that MCH treatment increases MAP2 expression both in at the transcription (*P* < 0.05) (Fig. 8A) and protein (*P* < 0.05) (Fig. 8B, C) level. *Bcl2* plays a crucial role in maintaining the delicate balance between cell survival and apoptosis. Our study revealed that infection led to a downregulation of *Bcl2* transcription levels (*P* < 0.01), whereas treatment with MCH restored the transcription levels of *Bcl2* (*P* < 0.05) (Fig. 8A).

## Discussion

Hormones, a class of chemical substances secreted by the endocrine system, play crucial roles in regulating various physiological processes [32]. The hypothalamus, a key region, enables the nervous system to regulate the endocrine system and is, in turn, influenced by it. The interplay between hormones and central nervous system diseases is significant, as hormones regulate neuronal activity, synaptic transmission, and neurotransmitter release, thereby influencing the central nervous system’s function [33]. In noninfectious central nervous system diseases such as intracerebral hemorrhage (ICH), PD, AD, and narcolepsy, hormones demonstrate differential expression and regulatory impacts. In ICH mouse models, there is a decrease in endogenous OXT levels and an increase in oxytocin receptor (OXTR) expression [34]. Melatonin, neural stem cell transplantation, or their combination have shown improvement in rotational behavior in PD mouse models [35]. Follicle-stimulating hormone (FSH) acts directly on hippocampal and cortical neurons, accelerating amyloid-beta and Tau protein deposition and impairing cognitive function in AD mice [36]. Moreover, a significant reduction in hypocretin-1 levels in the cerebrospinal fluid of narcolepsy patients has been observed [37]. Conversely, hormones also play a pivotal role in infectious central nervous system diseases. In Venezuelan equine encephalitis mouse models, melatonin administration delayed disease onset and prolonged survival [38]. Glucagon-like peptide-1 preserves neural structure by inhibiting lipopolysaccharide (LPS)-induced microglial inflammation [39]. In bacterial meningitis, serum procalcitonin levels increase abnormally, serving as a reliable marker to differentiate between bacterial and viral meningitis [40]. In the LPS-induced infectious disease model, there was a significant increase in chemokines (CCL2), proinflammatory cytokines (IL1β, IL6, TNFα), and a marked decrease in MCH [23]. Administration of CCL2 in mice reduced electrical activity of MCH neurons, influencing MCH release, mediating weight loss, and decreasing food intake, which could be reversed by inhibiting CCR2.

The functions of hormones extend beyond singular roles. OXT, for example, not only plays a role in birthing but also in social behavior, appetite control, and cognition. In patients with depression, plasma OXT levels are significantly negatively correlated with anxiety symptoms [41]. In the ventromedial hypothalamic nucleus (VMH), increased OXT induces satiety in rats, significantly enhancing energy expenditure and physical activity [42]. Intranasal OXT administration can mitigate sensory–motor dysfunction and cognitive decline due to ICH, reducing inflammation via the OXTR/p-PKA/DRP1 signaling pathway [34]. FSH impacts fat reduction, thermogenesis, and serum cholesterol, and influences Alzheimer's disease-like phenotypes through the neuron C/EBPβ–δ-secretase pathway [36]. MCH, known for stimulating feeding [43], has comprehensive research in energy regulation and metabolic homeostasis. Studies show that intracerebroventricular infusion of MCH and mice overexpressing MCH exhibit increased appetite and weight gain [44, 45]. Conversely, inhibiting MCH or MCHR1 leads to weight loss in mice [46, 47]. The hypothalamic control of appetite involves complex interactions. Paraventricular nucleus neurons releasing thyrotropin-releasing hormone indirectly inhibit MCH neurons by increasing GABAergic synaptic inhibition [48], while arginine vasopressin and oxytocin can directly activate MCH neurons, inducing food intake [49]. Additionally, MCH neurons inhibit the electrical activity of pro-opiomelanocortin (POMC) neurons in the arcuate nucleus, known for inducing negative energy balance. MCH also reduces energy expenditure by decreasing thermogenesis in BAT [50]. Mice with ablated MCH neurons demonstrate increased energy expenditure, decreased fat mass, and elevated expression of UCP1 and cytochrome c oxidase 4 (COX4) in BAT [51]. Furthermore, MCH promotes lipid storage in white adipose tissue and the liver, impacting energy storage. Chronic central infusion of MCH increases food intake and body weight, influencing lipid storage and mobilization in white adipose tissue, and triggering lipid accumulation in the liver [45]. The appetitive and lipogenic effects of MCH have potential therapeutic applications in diseases characterized by chronic muscle or fat mass loss, such as sarcopenia or cancer cachexia [52]. MCH neurons also project to brain regions associated with learning and memory. Injecting MCH into the hippocampus and amygdala of rats improves memory performance [53], and its infusion into rat hippocampal sections enhances evoked responses in the dentate gyrus [54]. MCH increases hippocampal synaptic transmission through an *N*-methyl-d-aspartate receptor-dependent pathway, lowering the threshold for long-term potentiation generation [55]. In PD models, MCH treatment increases primary dopaminergic neurons and rescues neuronal cell death induced by MPTP or 6-OHDA [22]. The neuroprotective effects of MCH on dopaminergic neurons involve PI3K/Akt, PKA/CREB, and MEK/MAPK pathways [22]. Intranasal MCH administration ameliorates levodopa-induced dyskinesia in PD mice, a process blockable by MCH receptor antagonists [56]. Striatal transcriptome analysis indicates *Tac2*, *Cartpt*, and *Lcn2* as potential mediators of MCH’s antimotor dysfunction effects [56]. In our research, the antidyskinesia effect of MCH was also observed in the mice infected by AC, but the mechanism needs to be further explored.

In our study, significant changes in *Pmch* were observed through transcriptome analysis of the whole brain in mice. This was further confirmed by RT–qPCR, which indicated reduced *Pmch* levels in the infected group. Mice infected with AC showed a progressive weight loss of 18–25% at 21 dpi, exceeding the diagnostic criteria for severe malnutrition (more than 10% loss of body weight within 6 months) [57]. In cases of infectious, inflammatory diseases, or cancer, substantial weight loss and the progressive loss of adipose tissue and skeletal muscle often imply a poor prognosis [58]. Our results showed that AC infection led to significant weight loss in mice. This was attributed to reduced energy intake (lowered food consumption), decreased energy storage (increased consumption of sugar and white adipose tissue), and gastrointestinal dysfunction (delayed gastric emptying and reduced small intestinal propulsion). Intranasal administration of MCH in these mice improved survival and mitigated body weight and white adipose tissue loss. Neurological function scores, evaluating various aspects such as voluntary movement, limb symmetry, climbing, balance, tactile sensation, and nociception indicated that the infected mice exhibited delayed locomotion, limb incoordination, and difficulty in maintaining balance. The MCH-treated group, however, showed significant improvements. In the Y-maze spontaneous alternation test assessing spatial memory, the MCH group demonstrated a higher alternation rate than the infected group. The NOR test, used to assess cognitive function, indicated that the infected group did not differentiate between new and old objects and exhibited significant locomotor impairment, as evidenced by reduced movement distance and speed. This impairment, also observed in Y-maze tests, was reversed by intranasal MCH administration. Pole tests are widely used to assess motor dysfunction in rodents. The procedure required mice to grip and manipulate below the bottom of a pole to assess the animal’s motor coordination. The MCH group demonstrated a shorter time than the infected group. These behavioral experiment results suggest that MCH improved cognitive function in infected mice to some extent, particularly in ameliorating motor deficits. Cognitive, memory, and motor signaling pathways are routed through synapses. Normal synaptic function depends on the stable expression of synaptic proteins. By quantifying the expression levels of synaptic-associated proteins (PSD95, SYP) and neuronal cytoskeleton protein MAP2 in cortical regions, we have observed that MCH exhibits a protective effect against the depletion of synaptic proteins. Additionally, MCH induces an upregulation in the transcription of anti-apoptotic protein *Bcl2*. Considering the observed benefits of MCH in enhancing survival, alleviating malnutrition, and reducing dyskinesia, we plan to further explore its potential as an adjunct therapy in AC treatment.

Therapeutic approaches stemming from the MCH system can be classified into two categories: direct MCH supplementation and the utilization of MCHR1 antagonists. While no clinical trials have been identified for direct MCH supplementation, more extensive clinical trials have been conducted for OXT, a related peptide. In a controlled clinical trial involving individuals with schizophrenia, participants were administered nasal OXT (24 IU) or saline via a spray bottle. Subsequent magnetic resonance imaging (MRI) scans revealed improved social cognitive functioning following OXT application [59]. Notably, OXT comprises only nine amino acids, which may partially account for its extensive research focus [60]. Although MCHR1 antagonists have demonstrated efficacy in mouse models of obesity [61], their performance in clinical trials has been less remarkable [62]. Addressing central nervous system (CNS) exposure remains a challenging aspect that requires further exploration.

While our study presents valuable insights, it is important to acknowledge certain limitations. Firstly, we did not investigate the potential relationship between inflammation and MCH. Secondly, the inclusion of MCHR1 antagonists in our research could have bolstered our evidence base.

## Conclusions

In our study, we explored the reasons for the apparent weight loss in mice and underscored the positive impact of MCH on energy balance and motor capabilities in a mouse model of AC infection. These findings not only contribute novel insights into the functionality of MCH but also provide a theoretical foundation for the development of therapeutic drugs for AC treatment.

### Supplementary Information

**Additional file 1: Figure. S1.** Experimental design and grouping. AC, *Angiostrongylus cantonensis*; dpi, days post-infection; MCH, melanin-concentrating hormone. RT-qPCR, real-time quantitative PCR.

**Additional file 2: Figure. S2.** GO enrichment analysis of differential genes induced by AC infection. (A) Biological processes. (B) Molecular functions. (C) Cellular components.

**Additional file 3: Figure. S3.** AC infection causes gastrointestinal motility disorders in mice. (A) Macroscopic images of the mouse intestines. (B) Statistics of first pellet staining for fecal expulsion time. (C) Statistics of fecal pellet frequency within 8 h. (D) Statistics of fecal pellet output within 8 h. (E) Statistics of gastric residual rate. (F) Statistics of intestinal propulsion rate. (G) Statistics of fecal moisture content. Data are presented as mean ± SD, *n* = 5. Compared to the Control group, statistical significance is indicated as *P < 0.05, **P < 0.01. dpi, days post-infection; TGITT, total gastrointest inal transit time.

**Additional file 4: Figure. S4.** Transcription levels of lipid metabolism-related enzyme genes in mouse gWAT. Data are presented as mean ± SD, *n* = 6. Compared to the Control group, statistical significance is indicated as *P < 0.05, **P < 0.01, ****P < 0.0001. *Fasn*, fatty acid synthase; *Hsl*, lipase, hormone sensitive; *Pparg*, peroxisome proliferator activated receptor gamma; *Acaca*, acetyl-Coenzyme A carboxylase alpha; *Cpt1α*, carnitine palmitoyltransferase 1a, liver; *Lpl*, lipoprotein lipase; *Atgl*, Patatin-like phospholipase domain-containing protein 2; *Lipa*, lysosomal acid lipase A; *Ucp1*, uncoupling protein 1; *Pgc1α*, peroxisome proliferator-activated receptor gamma coactivator 1-alpha; *Plin*, perilipin 1; *Cebpa*, CCAAT/enhancer binding protein alpha.

**Additional file 5: Table S1.** Primers for RT–qPCR (mice). For abbreviations, see Fig. 1, Fig. 8 and Fig. S4.

## Data Availability

All data supporting the conclusions of this study are included in the article.

## Abbreviations

Aβ: Amyloid β-protein
AC: *Angiostrongylus cantonensis*
BAT: Brown adipose tissue
BCL2: B cell leukemia/lymphoma 2
Cartpt: Cocaine- and amphetamine-regulated transcript protein
Ccl8: C–C motif chemokine ligand 8
*Chi3l3*: Chitinase-like 3
COX4: Cytochrome c oxidase 4
CREB: CAMP-response element binding protein
dpi: Days post infection
FITC: Fluorescein Isothiocyanate
FSH: Follicle-stimulating hormone
GEO: Gene Expression Omnibus
GLU: Glucose
GSK3β: Glycogen synthase kinase-3β
gWAT: Gonadal white adipose tissue
HDL: High-density lipoprotein
H&E: Hematoxylin and eosin
iWAT: Inguinal white adipose tissue
LDL: Low-density lipoprotein
LPS: Lipopolysaccharide
MAP2: Microtubule-associated protein-2
MAPK: Mitogen-activated protein kinase
MCH: Melanin-concentrating hormone
MCHR1: Melanin-concentrating hormone receptors 1
MWM: Morris water maze
NOR: New object recognition
Oxt: Oxytocin
PBS: Phosphate-buffered saline
Pmch: Pro-melanin-concentrating hormone
POMC: Pro-opiomelanocortin
PSD95: Postsynaptic density protein 95
RT–qPCR: Real‑time quantitative PCR
SD: Standard deviation
SYP: Synaptophysin
TC: Total cholesterol
TG: Triglycerides
