# Supplementary material for: Angiostrongylus cantonensis induces energy imbalance and dyskinesia in mice by reducing the expression of melanin-concentrating hormone
Source: Parasit Vectors. 2024 Apr 23;17:192. doi: 10.1186/s13071-024-06267-9 (PMC11036757; doi:10.1186/s13071-024-06267-9)
Supplement: Supplementary file 5 — Additional file 5: Table S1. Primers for RT–qPCR (mice). For abbreviations, see Fig. 1, Fig. 8 and Fig. S4. [file 13071_2024_6267_MOESM5_ESM.docx]

Table S1 Primers for qPCR (mice).

| Gene | Forward primer sequence | Reverse primer sequence |
| --- | --- | --- |
| *Pmch* | AGAGATTTTGACATGCTCAGGTGTA | GGTATCAGACTTGCCAACATGGT |
| *Fasn* | AGGTGGTGATAGCCGGTATG | TGGGTAATCCATAGAGCCCAGT |
| *Hsl* | TGTGGCACAGACCTCTAAAT | GGCATATCCGCTCTC |
| *Ppary* | TTTTCAAGGGTGCCAGTTTCGATCC | AATCCTTGGCCCTCTGAGAT |
| *Acaca* | GCCTCAGGAGGATTTGCTGT | AGGATCTACCCAGGCCACAT |
| *Cpt1a* | CTACATCACCCCAACCCATATT | GATCCCAGAAGACGAATAGGTT |
| *Lpl* | GAAAGGGCTCTGCCTGAGTT | TAGGGCATCTGAGAGCGAGT |
| *Atgl* | GGAGACCAAGTGGAACATCTCA | AATAATGTTGGCACCTGCTTCA |
| *Lipa* | CCAAGTAGGTGTAGGCACCAG | CATCTTCCGGGAGTGGTCC |
| *Ucp1* | ATTCAGAGGCAAATCAGCTTTG | GTGTTTCTCTCCCTGAAGAGAA |
| *Pgc1a* | GGATATACTTTACGCAGGTCGA | CGTCTGAGTTGGTATCTAGGTC |
| *Plin* | GCTGTCTGAGACTGAGGTGG | CTCAGGGAGGTCTCCATCCA |
| *Cebpa* | CGCAAGAGCCGAGATAAAGC | CAGTTCACGGCTCAGCTGTTC |
| *Bcl2* | CCTGTGGATGACTGAGTACCTG | AGCCAGGAGAAATCAAACAGAGG |
| *Map2* | AGTGGCACCTCCACACCTAC | CGGATGATGGCAACTTTCTT |
| *Syp* | GAGCAGATTGCCATGTCTGA | AGTTCCACGATGAGCTGCTT |
| *PSD95* | TGAGATCAGTCATAGCAGCTACT | CTTCCTCCCCTAGCAGGTCC |
| *β-actin* | CAGGCATTGCTGACAGGATG | TGCTGATCCACATCTGCTGG |
